# Mr. Bradley, on Hernia

**Published:** 1806-07

**Authors:** James Bradley

**Affiliations:** Huddersfield


### *To the Editors of the Medical and Physical Journal.*

Gentlemen,

On lately seeing the last Edition of Mr. Wilmer's publication on hernia, in which he recommends a new mode of the taxis, or applying a steady and long continued pressure in the reduction of the strangulated part, I was much pleased, as it nearly corresponds with a method I several years since adopted, and which I am happy to see now partly sanctioned from so respectable a quarter. In the summer of the year 1803, I mentioned this practice to Mr. Hey of Leeds, in a series of letters privately addressed to that gentleman, on some subjects treated by him in his late publication. In one of these letters alluding to hernia, is the following passage: "Your method of taxis is to let the patient reduce the hernia himself. Permit me to say, that I have for a number of years past in my circumscribed practice, adopted a method almost similar to this, and which, consists in making a gentle and long continued pressure, to be applied uniformly to the strangulated part. I was led to this practice, from an idea that the contractile power of the surrounding stricture would by it be more effectually overcome." At the time of communicating this practice to Mr. Hey, I was convinced of its preference to the usual modes of reduction by the taxis, and that it required only the sanction of his, or some other respectable name, in order to make it known, and generally adopted among surgeons.

As few are the means of relieving those labouring under this complaint, without subjecting the patients to a hazardous and painful operation, any hint that may prove useful, is of much importance to the unhappy sufferers. Impressed with this consideration, I have ventured to communicate to you* all the information in this particular,

---

* This Letter was some months ago sent to the Editor of a periodical publication, and immediately addressed to Mr. Wilmer, whose eye, I suppose, it never met, on account of my not hearing from that gentleman.

cular, which a limited practice is capable of supplying; for it can only be by stating facts, and comparing the cases in which this method has been successfully tried, with others, in which it has failed, that its merit can be fairly estimated. I am, &c.

*Huddersfield, May* 12, 1806. JAMES BRADLEY.

---

The method I use of reducing an inguinal or scrotal hernia by the taxis, is as follows:

I lay the patient in a recumbent posture, with his shoulders and breech somewhat elevated, taking care to have the former rather higher than the latter. This position will bend the spine a little forwards, and relax the abdominal covering. After elevating his knees, and having pushed up the hernial contents towards the ring with my fingers, I apply the thick and under part of my hand, to the lowest part of the tumour. I now adjust the degree of pressure, which I purpose to be equally and uniformly continued during the operation. In order to ascertain this, the patient's feelings are to be consulted; for if the pressure excites the least uneasiness, it ought to be moderated, that which I have always used, has been little more than suspensory. If this force be sufficient to push the whole of the hernia over the brim of the pelvis, an achievment, I believe, seldom practicable; or, in case the hernia is confined to the groin, I then direct my pres sure obliquely upwards, and somewhat backwards, and in this situation I keep my hand, my arm resting all the while upon some firm easy body, for if this be not observed, it will soon be fatigued, and consequently it will be necessary to change hands, a circumstance that ought to be avoided as much as possible. When the inferior part of the tumour rests upon the pelvis, and the superior part elevates the integuments very considerably at the ring, notwithstanding the application of the above decribed force, I then direct my pressure on the under part of the tumour obliquely upwards, but not backwards, with one hand, and with the fingers of the other, press gently at its upper extremity.

Two objects, *a priori*, present themselves as necessary to be kept in view, namely, to dilate the opening, and abate irritation in the strangulated part. The first of these intentions is attained, in an eminent degree, by gentle and long-continued pressure, in such a manner, as to apply equally and uniformly as possible, to every part of the surrounding rounding stricture. The second intention being that of diminishing irritation, concerns those parts within the ring, as well as those contained in the hernial sac. That irritation within the ring may be increased, seems sufficiently obvious. We will suppose any part of the abdominal *viscera*, for instance, a portion of the intestine pushed down thro' the aperture, and strangulated. In order to reduce this, the patient's body is either inverted, or at least his breech is elevated higher than his shoulders. Whoever will reflect on the facility which an unobstructed hernia passes up into the abdomen, when the body is in a supine posture, must be convinced, under such treatment, a considerable weight will be suspended by the part, at the point of strangulation; and as long as this suspension continues, an increase of irritation must necessarily ensue. Will not the position, in which this new mode of the taxis places the body, obviate this evil, and be equally as favourable for reduction, on a supposition that the stricture is removed? Will any other position be more favourable for removing the stricture, with as few inconveniences attached to it? Will hernia recede before the stricture is overcome? Will not the irritation at the ring, necessarily occasioned as above explained, far more than counterbalance any probable chance of the hernia receding from the gravitating power of the abdominal contents?

I think that no possible injury can be done to the hernial contents, according to the practice recommended in this mode of the taxis. It appears, at least to me, to possess, on the principle of pressure, all the advantages of the weight recommended by Mr. Wilmer, without any of its objections. According to the former method, the degree of pressure can be more properly ascertained, and more equally and directly applied to the stricture, without increasing irritation in the parts confined to the hernial sac, and which pressure by a hard body can scarcely fail to produce; for if any part of the hernial contents rests upon the brim of the *pelvis*, such a substance as the weight alluded to must necessarily expose them to injury.

It is now nearly thirteen years since I first practiced this new method of the taxis, and the subjoined cases of strangulated herniæ are the whole I have met with in that period. I have, however, much to regret, that in three of these cases, viz. 2d, 4th, and 6th, I took no notes, therefore have depended on my memory for every circumstance I have related respecting them; and although subject to some omissions, yet what I have communicated, I be-

I believe may be depended on, especially as to the certainty of the complaint; and of the duration, the mode, and result of the operation, I can speak decidedly. In all of these cases, the degree of pressure employed, has been uniformly the same, excepting case 7, in which, on account of the hernia being painful to the touch, rather less pressure was practiced. On succeeding in the first case, I was at a loss how far to ascribe the reduction to pressure, as this was combined with other means; I therefore resolved, in future, as far as was consistent with propriety, to give the taxis a fair trial, before I adopted any other remedies. In case 7th, the cathartic solution was administered from evident symptoms of *enteritis*; and here, as well as in case 1st, where this medicine was adminstered, I could not perceive any of those unpleasant effects ascribed to purgatives in general. The small quantity taken into the stomach, not proving sufficient to increase the disorder of that organ, and the position in which the patients were placed, might tend, perhaps, in some measure, to obviate any increased distress arising in that quarter. I gave this medicine, not with a view of obtaining any laxative effects, but as a cooling sedative, calculated to abate irritation in the first passages, under the circumstances of a quick pulse, considerable thirst, and great pain in the abdomen. I was led to adopt this remedy in preference, from observing its good effects in cases of enteritis, and in obstinate constipations of the bowels attended with colic, which I have seen it frequently remove, before any laxative effects have been produced. In those cases of strangulated herniæ, where the pulse is languid, and the vital energy considerably impaired, I think this medicine capable of doing harm.

In three cases of scrotal herniæ, accompanied with pain and irritation in the tumour, and which the patient's usual efforts failed to reduce, I succeeded in returning in less than fifteen minutes, according to the above method.

### CASE I.

At five o'clock in the morning of the 3d of December 1793, I was sent for to Mary Womersley (now Mary Ellis) nigh this place, aged 29 years. I found her labouring under symptoms of a strangulated femoral hernia. A little time previous to this, she had consulted me on account of a small tumour of the hernial kind, under Poupart's ligament. For this a steel truss was recommended, which I believe she wore, till she thought it no longer necessary.

About

About four o'clock in the afternoon of the preceding day, as she was straining on the close-stool, in consequence of a troublesome tenesmus, she suddenly felt a pain in the part, where the tumour had been lately situated, succeeded by sickness, vomiting, and frequent attempts at micturition. The vomiting had continued at intervals all the night, and generally was preceded by hiccuping, especially on taking any liquid into the stomach. She had had no stool since the strangulation, though she had made several attempts in consequence of the tenesmus. She complained of pain in the tumour, and all over the abdomen; but on examining the latter, it neither appeared tense nor swelled. She had some thirst, but her tongue was very little furred, and her pulse no higher than 87. I gave her 15 grains of the *sal amarus*, dissolved in a table spoonful of cold water, and ordered it to be repeated every half hour. A stupe was also wrung out of the aq. litharg. acet. comp. and applied to the tumour, and on this I pressed gently with the under part of my hand upwards, and somewhat downwards, for the space of an hour and four minutes, when I perceived the hernia to recede into the abdomen, and immediately followed by a guggling noise of the bowels.

### CASE II.

In the year 1794, I was desired to see John Booth, aged 32 years, who had been afflicted with an inguinal hernia for some time, and which now had become strangulated. I found him suffering great pain, especially in the tumour, and lower part of the abdomen, and that he had vomited several times. The hernia, instead now of being inguinal, was pushed down to, and rested upon the *epididimis*, and exhibited a semilunar appearance. I gently pushed up the tumour towards the ring, and kept my hand upon it so as to press gently, but without affording any increased pain, and in twenty-eight minutes the hernia returned without any noise, or other indication of its reduction.

### CASE III.

Robert Bailey, a stout athletic man, aged 65 years, and who had been subject to a scrotal hernia for several years, had the misfortune May 2, 1795, whilst lifting a piece of timber, to bring on strangulation. I found him complaining of great pain all over the abdomen, attended with sickness and vomiting, which had existed for nine hours previous to my seeing him. His pulse was full, but not hard, and at 85, and his thirst was also inconsiderable, and tongue very little furred. He had one stool immediately after the strangulation, and had made water several times. I pushed up the hernial contents towards the abdominal aperture, and retained them there according to the preceding directions, for the space of two hours and three quarters, changing hands however in this period three or four times. I now found it adviseable to desist from this practice, on account of the inflammatory symptoms increasing; as his pulse was risen to 95, and more depressed; his thirst was also more considerable, and the pain in the abdomen increased. A glyster consisting of a drachm and a half of tobacco boiled in a pint of water for ten minutes was injected. This excited great sickness, and caused him to retch several times. After the effects of the glyster had apparently subsided, he complained of a farther increase of pain in the abdomen, and his pulse had risen to 116. His thirst was also considerably augmented, and his tongue more parched and dry. He was restless, with great anxiety at the *præcordia*. Under these circumstances, on the operation being had recourse to, it was found that about eight inches of intestine was contained in the hernial sac inflated, and highly inflamed, but without exhibiting any marks of gangrene. He expressed great relief from the operation, and soon afterwards fell into a comfortable sleep; notwithstanding this, when he awoke he got out of bed, and drank so immoderately of malt liquor in the absence of his wife as to intoxicate himself, and which caused him to fall down, and lie for some hours on a damp floor; and so addicted was this unfortunate man to inebriety that he no sooner became somewhat sober, than he repeated the noxious potion, which necessarily produced consequences that proved fatal.

### CASE IV.

In the autumn of the year 1795, I was called to a person of the name of Joseph Brooke, upwards of thirty years of age, who I found had been labouring under a strangulated scrotal hernia for many hours. I gently pushed up the hernial contents to the abdominal ring, and retained them in that situation for the space of two hours, pressing gently on them the whole time. From the state of his case, it was now found adviseable to desist from this method, and to apply more force, in order to reduce the swelling; on this also failing, most of the means recommended in such cases were put in practice, but all to no purpose. The operation was proposed, but not acceded to, I therefore reluctantly abandoned the poor fellow to his fate.

CASE

### CASE V.

In the month of September 1796, on passing through Dogley Bar, near this place, I was desired to see a male child of George Marsden, a resident there, aged eleven months, and who, as was supposed, was dying of teething. The parents informed me that twenty-fours hour before I saw the child it was pretty well, but in that interval had vomited at least every half hour. The child lay quite still, only now and then turned its head, and frequently moaned. The *tunica adnata* and countenance were extremely pallid, which, together with cold sweats, indicated great sickness. The patient had not been at stool for the last thirty hours. This circumstance induced me to turn up its cloaths, in order to examine the abdomen, when I discovered a scrotal hernia, and in a strangulated state. The mother informed me, that the swelling she had first discovered about three days subsequent to the birth of the child; and for the last two days it had not receded, and corresponded in appearance with what it now presented. Being satisfied as to the cause of the little sufferer's distress, I passed up the swelling towards the ring, and retained it there with my fingers, and in less than five minutes the hernia returned with a guggling noise.

On the reduction of the hernia the child's countenance immediately changed, for instead of a deadly or pallid hue, a healthy and bloomy suffusion ensued. It became at the same time lively, and took the breast in a few minutes, and soon after this had a stool.

No doubt such a case as this very rarely occurs; but I think every medical man when he is called to an infant, who cannot describe its own feelings, ought, where the circumstances of vomiting and costiveness occur, to examine the lower part of the belly. This he may do without betraying any suspicion.

### CASE VI.

Joshua Stocks, upwards of fifty years of age, and who for several years had been troubled with a scrotal hernia, was in the year 1801, seized with symptoms of strangulation. I saw him six hours after he was first attacked. He complained of great pain all over the abdomen, attended with sickness and vomiting. I practiced the mode of pressure above described, and in twenty-five minutes the hernia returned into the abdomen.

Here there appeared to be a considerable portion of the abdominal contents down in the hernial sac, and in this respect similar to the case of *Bailey* and *Brooke*, which afforded so much trouble; I therefore anticipated a great deal of obstinacy in its reduction, but found myself agreeably disappointed.

### CASE VII.

Susannah North, aged 73 years, was seized with symptoms of enteritis, viz. with rigors succeeded by heat, and great pain in the belly, attended with costiveness. These symptoms continued all the remainder of the 17th day and following night, till the morning of the 18th, when vomiting succeeded, and continued frequently at intervals till one o'clock of that day, when I found her in great distress. Her pulse was at 103, tongue much furred, thirst very considerable; she was restless, frequently sick, and vomited at intervals, especially on taking any thing on the stomach, and generally was preceded by some hiccuping. The abdomen was considerably distended, and all over painful to the touch, especially the lower part of the hypogastric region. On questioning her, she acknowledged having a swelling, which I found to be a femoral hernia about the size of a large walnut, and which she said had appeared at times for some years past, but without affording her any trouble excepting now and then a little painful, and always the largest when she had pain in her bowels, which she was subject to. I gave her twenty grains of the *magnesia vitriolata* every twenty minutes, and immediately applied a small compress of linen to the tumour, and pressed gently on this upwards and a little downwards for the space of eighteen minutes, when I perceived the tumour to decrease, and a slight borborygmi of the bowels to ensue. All the symptoms were now relieved, and she became disposed to sleep, I therefore left her, and again visited her in the evening at nine o'clock, and found her much better. The abdomen was softer, and less painful to the touch; her pulse was more elevated, and down at 94, and her tongue was less furred, but her thirst was still considerable. Had yet no stool, and had only made water once since the hernia was replaced, and indeed had only made water once from the time of strangulation to the time of reduction. In a few hours after her bowels became opened, and the next day was so well as to require no farther attendance.

